# Four novel *RS1* gene mutations in Polish patients with X-linked juvenile retinoschisis

**Published:** 2012-12-13

**Authors:** Anna Skorczyk, Maciej R. Krawczyński

**Affiliations:** 1 Department of Medical Genetics, Poznan University of Medical Sciences, Poznań, Poland; 2 Center for Medical Genetics “*Genesis*,” Poznań, Poland

## Abstract

**Purpose:**

To determine the clinical features and to identify mutations in the retinoschisis gene (*RS1*) in ten patients with X-linked retinoschisis (XLRS).

**Methods:**

Ten male patients from nine Polish families were included in this study. Ophthalmologic examinations, including optical coherence tomography (OCT) and full-field electroretinography (ERG), were performed in all affected boys. The entire coding region encompassing six exons of the *RS1* gene was amplified with PCR and directly sequenced in all the patients.

**Results:**

All affected individuals showed typical retinoschisis signs and symptoms, and all appeared to have a mutation in the *RS1* gene. Seven different mutations were identified, including two novel missense substitutions: c.176G>C (p.Cys59Ser), c.451T>A (p.Tyr151Asp); one novel nonsense substitution: c.218C>A (p.Ser73*); and one novel frameshift mutation: c.354_355delCA (p.Asp118Glufs*2). We also found two missense substitutions that had been previously described: c.214G>A (p.Glu72Lys) and c.626G>T (p.Arg209Leu) and one known splice site mutation in intron 5: c.522+1G>T (IVS5+1G>T).

**Conclusions:**

This study provides the first molecular genetic characteristics of patients with juvenile retinoschisis from the previously unexplored Polish population. We investigated the molecular background of XLRS in ten boys. The present study reports for the first time four novel mutations, including two missense substitutions, one nonsense substitution, and one frameshift deletion. One of these substitutions and 2-bp deletion created stop codons. Moreover, we described three substitutions that had been previously reported (one is a splicing mutation). Further genetic characterization of Polish patients with XLRS will be helpful in understanding the worldwide spectrum of *RS1* mutations. Despite the mutation heterogeneity found in a small group of our patients, they presented a relatively uniform clinical picture. Identifying the causative mutation is helpful in confirming diagnosis and counseling, but cannot provide prognostic data.

## Introduction

X-linked juvenile retinoschisis (XLRS; RS, MIM# 312700) is one of the leading causes of juvenile macular degeneration in men [1] and affects 1 in 5,000 to 1 in 25,000 men worldwide [2]. XLRS is caused by mutations in the *RS1* gene on chromosome Xp22.2 [3]. The gene spans 32.4 kb of genomic DNA and encodes a 224-amino acid extracellular protein known as retinoschisin. Retinoschisin is secreted from photoreceptor cells of the outer retina and bipolar cells of the inner retina. This protein plays a crucial role in the cellular stabilization and organization of the retina [4,5]. The RS1 protein contains three domains: a signal sequence encoded by exons 1 and 2 (amino acids 1–23), an Rs1 domain encoded by exon 3 (amino acids 23–62), and a discoidin domain encoded by exons 4–6 (amino acids 63–219). In addition, a five-amino-acid segment is present at the C-terminal side of the discoidin domain [6].

More than 190 mutations in the *RS1* gene have been reported to date in patients with XLRS (Leiden Open Variation Database, LOVD version 2.0, Build 31). The majority are nucleotide substitutions resulting in amino acid changes, alterations of splice site sequences, or activations of cryptic splice sites, although deletions and insertions have been also found [7]. Most of the missense variants that constitute the largest group of *RS1* gene mutations were identified within the highly conserved region of the gene that encodes the discoidin domain of the protein. This domain is thought to be involved in cell-to-cell interactions on membrane surfaces [8,9] and is crucial for RS1 protein function [2]. Three retinoschisis mouse models have been developed, and male hemizygous mice presented retinal pathology similar to the human disease at an early age [10-12].

X-linked juvenile retinoschisis belongs to the group of vitreoretinal dystrophies and causes progressive impairment of vision with variable degree of severity. Usually the clinical phenotype includes reduced visual acuity, strabismus, or vitreous hemorrhage, and manifests between 5 and 10 years of age. XLRS can also lead to retinal holes and detachment [13].

## Methods

### Clinical studies

**Figure 1:**
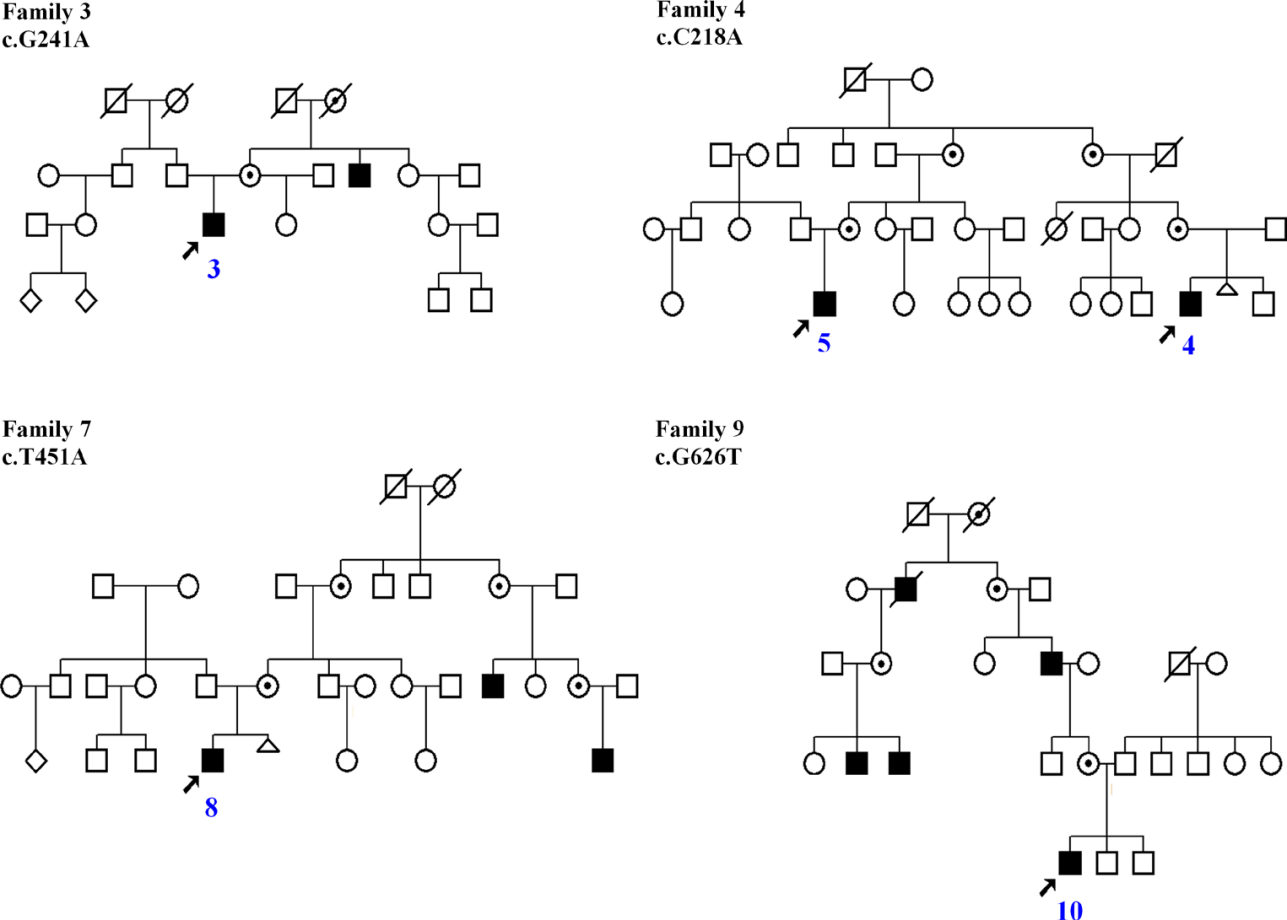
Pedigrees of families with X-linked juvenile retinoschisis with positive family history. Black squares represent affected boys. Circles with black dots denote obligate carriers. Arrows point to probands. Slashed circles and squares are deceased family members. Blue digits indicate the patients’ numbers.

Ten male patients aged 5–17 exhibiting the clinical features of juvenile retinoschisis, from different regions of Poland, were examined. At the time of recruitment the general state of health of all our patients was good. Eight came from unrelated families, while two affected boys were identified as second cousins (Figure 1). Voluntary informed consent was obtained from the parents of all patients as they were all under age at the time of examination. Ophthalmologic examinations, including optical coherence tomography (OCT) and full-field electroretinography (ERG), were performed in all affected boys.

### Detection of mutations

Peripheral blood samples were obtained from probands only. Blood was collected into tubes containing EDTA and stored in a refrigerator until DNA isolation was performed. Genomic DNA was extracted from leukocytes using the standard salting-out procedure [14]. The entire coding region comprised of six exons and the flanking intronic regions of the *RS1* gene (GenBank NG_008659.1) was amplified with PCR using standard primers. The sequences of the primer sets and the length of the PCR products are shown in Table 1. For all six amplified fragments, the following PCR conditions were applied: 95 °C, 3 min (preliminary denaturation); 40 cycles of denaturation at 94 °C for 30 s, annealing for 30 s with temperature starting from 63 °C, decreasing to 55 °C (touchdown PCR −0.2 °C per cycle), elongation at 72 °C for 45 s; and the final synthesis at 72 °C, 10 min. PCR was run in a Mastercycler pro thermocycler (Eppendorf, Hamburg, Germany) using HiFiTaq polymerase (Novazym, Poznań, Poland).

**Table 1:** Primer pairs and size of PCR products used to screen *RS1*gene for mutations

Primer symbol	Primer sequence (5′-3′)	Size
F1	5′ ACTTAATCCCCTGCTCCTGG 3′	208
R1	5′ TCAGGCTATATTCCTATTTATCAACG 3′	
F2	5′ TAGCTTCTTAGCATCTGCGG 3′	661
R2	5′ TGTGCTACAGTCACCATCACC 3′	
F3	5′ GCTGTGTGTATTGAGGCTGG 3′	451
R3	5′ TGGAGAAAACCCGCATTAAC 3′	
F4	5′ AAAGCAGATGGGTTTGTTTTG 3′	302
R4	5′ GGCCACGCTGGTAGAGAG 3′	
F5	5′ CTCGAGAGCCAGCACCTG 3′	337
R5	5′ GGGACAGGAGGGGAAGTC 3′	
F6	5′ GCTAGCTCCAGAAAGGAACC 3′	310
R6	5′ ATCTCGGTGGTGTGTGAGG 3′	

The amplicons were separated on 1% agarose gels stained with ethidium bromide. PCR products were directly sequenced using the Big Dye Terminator Cycle-Sequencing v3.1 Kit (Applied Biosystems, Foster City, CA) and run on an automated sequencer ABI 3130XL Genetic Analyzer (Applied Biosystems). The obtained sequences were verified by comparing them to the human genomic sequence of *RS1* (GenBank NG_008659) and screened for mutations. Identified variations were checked (whether they were described earlier or not) in LOVD for *RS1* and numbered according to Ensembl (ENSG00000102104). In silico analyses using SIFT software were performed to predict whether the novel missense mutations affect protein function. SIFT predictions are based on the degree of conservation of amino acid residues in sequence alignments derived from closely related sequences, collected through PSI-BLAST. The splice site prediction tool available on the Berkeley Drosophila Genome Project (BDGP) website was used to find possible 5′ and 3′ splice sites (Toolspice).

## Results

The clinical diagnosis was based on clinical data, OCT, and ERG results. All affected individuals showed typical juvenile retinoschisis signs and symptoms. Clinical results together with the molecular investigations are shown in Table 2. The age of onset was defined as either the patient’s age at which visual loss was first noted or the age documented in an ophthalmologic record of the first diagnosis. The age of onset of juvenile retinoschisis differed between individual patients and ranged from 1 to 11 years. In the majority of our patients, the first symptom of the disease was visual impairment, but two boys did not show any initial vision impairment. They were referred to the eye clinic due to vitreous hemorrhage.

**Table 2:** Clinical data and mutations identified in the patients with XLRS

No	Mutation	Age of onset (yrs)	First symptom	Actual VA	Funduscopic macular abnormalities	OCT	ERG	Other ophthalmologic symptoms	Affected relatives
1	c.176G>C (p.Cys59Ser)	1	strabismus	RE: 0.1 LE: 0.2	wheel-like cystic formation	typical for RS (intraretinal spaces, cysts)	-	RS of lower retina quadrants	none
2	c.176G>C (p.Cys59Ser)	2	vitreous hemorrhage	RE: 0.8 LE: 0.2	wheel-like cystic formation	typical for RS	scotopic b-wave decreased	myopic astigmatism, peripheral RS	none
3	c.214G>A (p.Glu72Lys)	1	strabismus, reduced VA	RE: 0.1 LE: 0.3	wheel-like cystic formation	typical for RS	scotopic b-wave decreased	-	maternal uncle
4	c.218C>A (p.Ser73*)	7	reduced VA	RE: 0.4 LE: 0.4	wheel-like cystic formation	typical for RS	scotopic b-wave decreased	-	second cousin
5	c.218C>A (p.Ser73*)	10	reduced VA	RE: 0.3 LE: 0.4	no macular reflex, pigment deposits	typical for RS	scotopic b-wave decreased	vitreal floaters	second cousin
6	c.218C>A (p.Ser73*)	7	reduced VA	RE: 0.8 LE: 0.2	pigment deposits	typical for RS	scotopic b-wave decreased	hyperopic astigmatism	none
7	c.354–355delCA (p.Asp118Glufs*2)	3	reduced VA	RE: 0.1 LE: 0.1	wheel-like cystic formation	typical for RS	-	initial Dg: CME	none
8	c.451T>A (p.Tyr151Asp)	6	reduced VA	RE: 0.5 LE: 0.6	wheel-like cystic formation	typical for RS	scotopic b-wave decreased	initial Dg: CME; central scotoma	2 affected relatives
9	c.522+1G>T	11	vitreous hemorrhage	RE: 1.0 LE: 0.6	wheel-like cystic formation	typical for RS	nonspecific (hemorrhage)	vitreoretinal proliferation, floaters	none
10	c.626G>T (p.Arg209Leu)	6	reduced VA	RE: 0.4 LE: 0.1	no macular reflex	typical for RS	scotopic b-wave decreased	RS of lower retina quadrants	4 affected relatives

Altogether, seven different mutations localized in exons 3–6 and intron 5 of the *RS1* gene were found in ten patients. Most of the mutations were localized within the region of the conservative discoidin domain (three mutations in exon 5, two substitutions in exon 4, one in exon 6, and one in intron 5), but we also found the mutation in exon 3. Among the identified mutations, there were two novel missense substitutions: c.176G>C (p.Cys59Ser), c.451T>A (p.Tyr151Asp), one novel nonsense substitution: c.218C>A (p.Ser73*), and one novel frameshift mutation: c.354_355delCA that introduces a stop codon (p.Asp118Glufs*2). We also found two previously described missense substitutions: c.214G>A (p.Glu72Lys) and c.626G>T (p.Arg209Leu), and one known splice site mutation in intron 5: c.522+1G>T. Five of these mutations were identified in one patient only, while the nonsense substitution: c.218C>A was found in three patients and the c.176G>C in two affected boys. The identified mutations, their type and localization within the *RS1* gene, and the number of affected patients are shown in Table 3.

**Table 3:** Identified *RS1* gene mutations

Mutation	Type of mutation	Effect of mutation	Localization	Number of patients	References
c.176G>C	missense	p.Cys59Ser	exon 3	2	this study
c. 214G>A	missense	p.Glu72Lys	exon 4	1	[7,17,18]
c.218C>A	nonsense	p.Ser73*	exon 4	3	this study
c.354_355delCA	frameshift	p.Asp118Glufs*2	exon 5	1	this study
c.451T>A	missense	p.Tyr151Asp	exon 5	1	this study
c.522+1G>T	splice site	abnormal splicing	intron 5	1	2
c.626G>T	missense	p.Arg209Leu	exon 6	1	LOVD

A novel missense substitution c.176G>C within exon 3 of the *RS1* gene (p.Cys59Ser) was found in two patients: 1 and 2. The mutation is located within the Rs1 domain of the protein. The in silico SIFT software analysis of the predicted influence of this novel substitution on protein function revealed that this amino acid change is deleterious, as the SIFT score was 0.01, which is categorized as a deleterious tolerance index score.

Patient 1 was a 5-year-old boy with strabismus detected in the first months of life, reduced visual acuity, and retinoschisis of the macula and both lower quadrants of the retina. OCT examination revealed numerous cysts and intraretinal spaces, typical for RS. The patient is the only child of healthy, unrelated parents, who have no ophthalmologic problems and negative family history.

**Figure 2:**
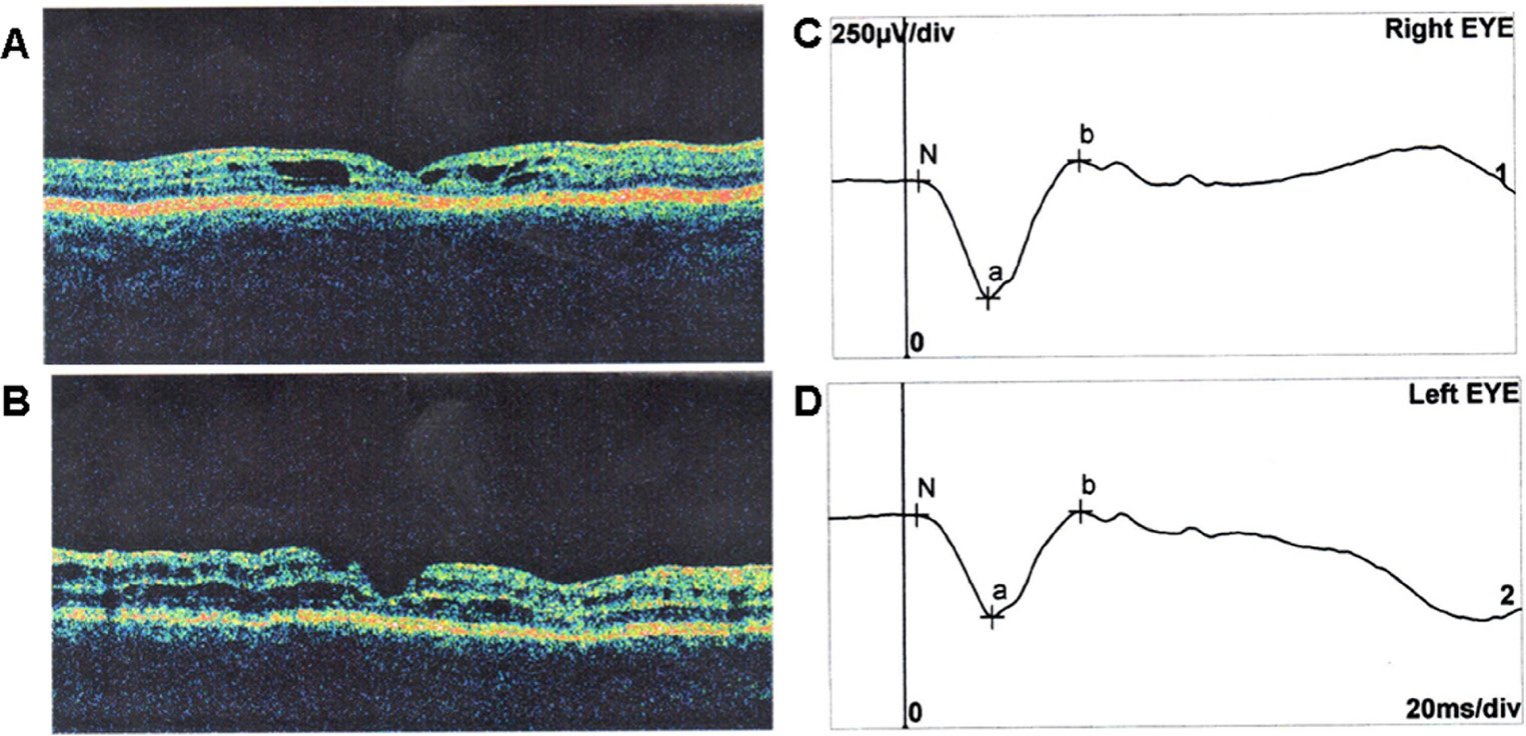
OCT and ERG results of patient 2. **A**: OCT image of the right eye showing foveal cystic retinoschisis. **B**: OCT image of the left eye showing foveal atrophy. **C**, **D**: Standard scotopic response of full-field ERG of the right and left eye showing decreased b-wave amplitude

Patient 2 was a 9-year-old boy. Initially, he suffered only from myopic astigmatism. At the age of 14 months, the boy underwent vitrectomy after a vitreous hemorrhage, which was the result of hitting the edge of a table. Ophthalmologic examination revealed characteristic wheel-like cystic formation across the macula region. OCT was typical for RS, and ERG examination revealed decreased scotopic b-wave (Figure 2). The parents, an older sister of the patient, and the other members of this family had no ophthalmologic problems. Both patients come from unrelated families (we have no information regarding their potential relationship).

A common substitution: c.214G>A was identified within exon 4 of the *RS1* gene (p.Glu72Lys) in patient 3. He was referred to the eye clinic due to reduced visual acuity and strabismus of the right eye. Ophthalmologic examination revealed characteristic wheel-like cystic formation. OCT was typical for RS, and ERG examination revealed decreased scotopic b-wave. The parents and the patient’s older half-sister did not show any eye problems, but his maternal uncle showed reduced visual acuity and similar retinal symptoms (Figure 1).

**Figure 3:**
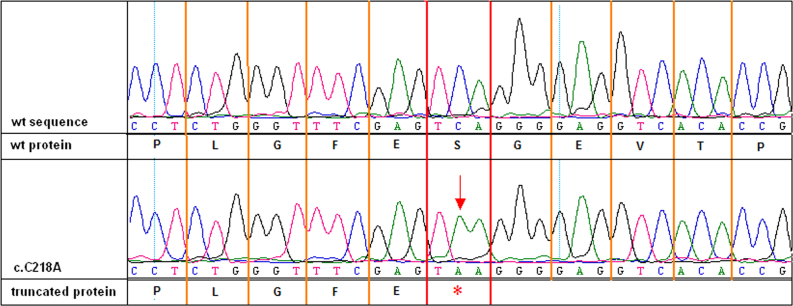
Chromatogram showing the c.218C>A (p.Ser73*) mutation. The upper part of the picture shows the wild-type nucleotide sequence and wild-type protein, while the lower one shows the nucleotide sequence with c.218C>A mutation and truncated protein.

A novel substitution: c.218C>A in exon 4 of the *RS1* gene (p.Ser73*; Figure 3) was found in three patients: 4, 5, and 6.

Patient 4 was referred to the eye clinic at the age of 7 due to the deterioration of his vision. Ophthalmologic examination revealed characteristic wheel-like cystic changes in the macula. OCT was typical for RS, and ERG findings showed decreased scotopic b-wave. The patient has an older brother, but neither his brother nor their parents showed any eye problems. After pedigree analysis, the patient appeared to be related to patient 5. The boys are second cousins (Figure 1).

Patient 5 manifested deterioration of vision at the age of 10. Ophthalmologic examination revealed a lack of macular reflex and pigment deposits. OCT was typical for RS, and ERG examination revealed decreased scotopic b-wave. The patient is the only child of healthy, unrelated parents, who have no ophthalmologic problems.

In patient 6, the loss of visual acuity was observed at the age of 7. Pigment deposits were observed in the macula. The OCT and ERG results were typical for retinoschisis. The patient has an older sister, but neither she nor their parents had any ophthalmological problems. This patient is not related to patients 4 and 5.

A novel deletion in exon 5: c.354_355delCA (p.Asp118Glufs*2) was found in patient 7. He was referred to the eye clinic due to deterioration of vision presented at the age of 3. Ophthalmologic examination revealed characteristic wheel-like cystic formation, and OCT was typical for retinoschisis. The initial diagnosis in this boy was cystoid macular edema (CME). Patient 7 is the only child of unrelated parents. His mother has no ophthalmologic problems, but myopia has been identified in the proband’s father and in several members of the father’s family.

A novel substitution: c.451T>A was identified in exon 5 of the *RS1* gene (p.Tyr151Asp) in patient 8. The mutation is located within discoidin domain of retinoschisin. The in silico analysis using the SIFT software revealed that this substitution has a serious impact on the protein function in the *RS1* gene, as the tolerance index score appeared to be highly deleterious (0.00). Patient 8 manifested reduced visual acuity at the age of 6. Ophthalmologic examination revealed characteristic wheel-like cystic formation. He also manifested a central scotoma, and the initial diagnosis was CME. Patient 8 is 10 years old. He is the only child of unrelated and asymptomatic parents, but similar eye problems were observed in several of the proband’s mother’s family members (Figure 1).

A substitution of guanine with thymine at nucleotide position 522 (c.522+1G>T) was identified in intron 5 (IVS5+1G>T) of the *RS1* gene in patient 9. BDGP splice site prediction software confirmed that this substitution changes the potential splice site encompassing GT nucleotides. Patient 9 is now 15 years old, and was referred to the eye clinic due to the vitreous hemorrhage in his right eye after a ball hit him at the age of 11. Then he had recurrent hemorrhages in his left eye. The results of all examinations were typical for retinoschisis; moreover, vitreoretinal proliferations were observed. The patient has a younger sister, but neither she nor their parents had any ophthalmologic problems.

A substitution c.626G>T in exon 6 of the *RS1* gene was detected in patient 10. This transversion results in an amino acid change from hydrophilic arginine to hydrophobic leucine at position 209 (p.Arg209Leu), which is located within the discoidin domain of the retinoschisin protein. The in silico analysis of the predicted influence of this novel substitution on protein function with the use of SIFT software revealed that this amino acid change may affect protein function, as the SIFT score was 0.02, which is categorized as an intolerant index score. Patient 10 manifested deterioration of vision at the age of 6. The patient has two younger brothers (7 years old and 9 months old), who had no ophthalmologic problems. The parents were healthy, too, but several members of the proband’s mother’s family presented the same symptoms as the proband (Figure 1).

## Discussion

This study is the first report of *RS1* mutations in Polish patients with retinoschisis. We report four novel mutations, including two missense substitutions, one nonsense mutation, and one frameshift deletion, as well as three previously described mutations identified in ten probands from Polish families. All affected individuals showed typical retinoschisis signs and symptoms, and all appeared to have a mutation in the *RS1* gene.

The missense substitution c.176G>C (p.Cys59Ser) within exon 3 of *RS1* identified in two unrelated patients (1 and 2) is described in the present study for the first time, although the substitution: c.175T>A affecting the same amino acid residue (p.Cys59Ser) has been reported [2,15]. Moreover, the substitution of guanine to adenine at the same nucleotide position (c.176G>A) changing amino acid cysteine to tyrosine was reported [16]. Cys59 is located outside the discoidin domain, but just before the start of this domain within the retinoschisis protein [7]. Wang and colleagues have observed the p.Cys59Ser mutation in COS-7 cells and investigated their intracellular processing and transport. The protein was successfully secreted from the cell, which suggests that this mutant may be dysfunctional [17]. Wu and Molday confirmed this suspicion based on their analyses of the expression, protein folding, disulfide-linked subunit assembly, intracellular localization, and secretion of the Cys59Ser mutant [6]. They concluded that Cys59 is not critical for the folding of the retinoschisin subunit but is responsible for intermolecular disulfide bonds that mediate retinoschisin oligomerization. The results of their studies indicate that Cys59 is one of many cysteine residues that are solely responsible for generating the 180-kDa disulfide-linked multimeric retinoschisin complex [6].

The substitution c.214G>A (p.Glu72Lys) identified in patient 3 is the most common *RS1* mutation known to date and has been reported 66 times [7]. This substitution has been described as the second most frequent mutation in Spanish patients [18], and as one of three founder mutations in Finnish patients [19]. This variant is listed in dbSNP (rs104894928), but no allele frequency data have been reported. The clinical significance of this substitution is described as “probably pathogenic allele.” This substitution is in a CpG mutation hot spot. The substitution changes amino acids at less conservative positions, but the properties of two amino acids involved in this mutation differ [3,19]. The acidic glutamic acid residue is changed to a basic lysine.

The nonsense mutation c.218C>A (p.Ser73*) was identified in three patients (patients 4, 5, and 6). All three boys came from different regions of Poland, but two (4 and 5) appeared to be second cousins. We did not identify a relationship between these two patients and patient 6. However, in the case of c.218C>A and c.176G>C substitutions, which were identified more than once in this study, we cannot exclude that some unknown familial relationships may exist. The substitution c.218C>A in exon 4 changes the actual cysteine codon to a stop codon and creates a truncated protein product that has only 73 amino acids of the C-terminal peptide losing most of the functional region (90% of the discoidin domain). This mutation has not been reported to date, but a nonsense mutation localized in the same region of the retinoschisin protein has been reported: c.238C>T (p.Glu80*) [20]. The functional analysis of c.238C>T substitution has not been performed, but it was suspected that p.Glu80* mutation might interfere with the retinoschisin octamerization and protein function.

The fact that we identified the c.218C>A substitution in three patients from three families (two of whom appeared to be related) may suggest that this substitution may be distributed wider in the Polish population and could be a founder mutation in the Polish population. Unfortunately, data regarding mutations identified in the *RS1* gene in the Polish population are not available, as there are no reports on this issue and no mutations have been submitted to the Leiden Open Variation Database for *RS1*. Some sequence alterations of the *RS1* gene are recurrent, for example, p.Glu72Lys, which was identified in this study, or two other missense substitutions (p.Gly74Val, p.Gly109Arg) found in 95% of affected individuals of Finnish heritage [21].

There was also another mutation with the effect of creating a stop codon discovered in our group of patients. It was the only frameshift mutation identified in this study: a 2-bp deletion in exon 5: c.354_355delCA identified in patient 7. This small frameshifting deletion creates a stop codon at position 119 of the protein (p.Asp118Glufs*2). Taking into account the nature of unique variants, about 40% are expected to represent true null alleles (i.e., nonsense mutations or frameshift mutations) and thus should not produce any functional retinoschisin protein [6]. To date, several nonsense mutations have been reported, for example, the p.Trp112* mutation that creates stop codon resulting in premature truncation of the protein product. This truncated protein could degrade rapidly leading to a null protein [22].

The substitution: c.451T>A was identified in one patient (patient 8). This transversion causes an amino acid change from tyrosine to aspartic acid (p.Tyr151Asp). Tyrosine at position 151 of the protein is a highly evolutionary conservative residue [3]. This fact together with the results of the SIFT analysis suggest that this amino acid change is likely to influence the protein function.

The splice site mutation c.522+1G>T, which was found in patient 5, was previously reported [2]. Moreover, the other substitution at this nucleotide position was described: c.522+1G>A [23]. The fact that intronic mutation c.522+1G>T includes guanine from the conserved GT splice donor dinucleotides, critical for splicing [2], together with the results of the in silico analysis (splice site prediction software), indicate that this substitution affects RNA splicing. However, this fact cannot be confirmed experimentally, as mRNA is expressed solely in the retina.

The substitution c.626G>T located within exon 6 of the *RS1* gene (p.Arg209Leu) was identified in patient 10. This transversion was described earlier in the Leiden Open Variation Database in one Hungarian patient with typical foveal retinoschisis, but the pathogenicity of this mutation was not analyzed, and LOVD says that the concluded pathogenicity of this variant is unknown. However, the substitution of hydrophilic arginine to hydrophobic leucine at position 209 (p.Arg209Leu) is very likely to result in a defective retinoschisin. This suspicion was confirmed by the results of the in silico analysis with the use of SIFT software. Moreover, similar substitution c.626G>A has been described in some reports [2,17,24-26].

Whatever the mechanisms of newly identified mutations are, future studies may provide some hope for therapeutic approaches for preventing retinal degeneration in patients with XLRS. As there is no current therapy for juvenile retinoschisis, the successful gene therapy performed in mice [27,28] gives hope that this therapy may be an option for treating patients with retinoschisis in the future. Gene therapy based on introducing a wild-type protein may be suitable for nonsense mutations but not for missense mutations that induce misfolded proteins. Young patients could benefit from this therapy before their retinal architecture has undergone degenerative changes [22].
